# Supplementary material for: Pharmaceutical enterprises integrity supervision strategy when considering rent-seeking behavior and government reward and punishment mechanism
Source: PLoS One. 2025 May 19;20(5):e0320964. doi: 10.1371/journal.pone.0320964 (PMC12088600; doi:10.1371/journal.pone.0320964)
Supplement: S1 File — (DOCX) [file pone.0320964.s001.docx]

Statistical data

All the data is available and within the manuscript, no supplement materials data.

According to the "2022 Drug Supervision and Administration Statistical Annual Data", regulatory agencies at all levels investigated and dealt with 514 cases of counterfeit and substandard drugs in 2022, with a total value of 430 million CNY. Based on this, this article sets the cost of drug production enterprises producing low-quality drugs for. According to the "National Medical Products Administration Department Budget for 2023", the market supervision and administration drug affairs budget at the beginning of the year is 450 million CNY. It is assumed that the cost of strict government supervision is. Based on relevant literature and combined with the realistic background, other parameters are assigned the following values: ,$W=25$,,,,,,,,,,,,.

**6.1 The Impact of Government Regulators on The Amount of Fines and Incentives Imposed on Drug Wholesale Enterprises**

Set,, the evolution process and results of the main strategies of the four-party game are shown in Figure 6.

**6.2 The Impact of Government Regulators on the Amount of Fines Imposed on Drug Production Enterprises and Third-party Testing Agencies**

Set,, the evolution process and results of the main strategies of the four-party game are shown in Figure 7.

**6.3 Impact of Reporting by Drug Wholesale Enterprises**

Set$z=\{0.0,0.5,1.0\}$, the evolution process and results of the main strategies of the four-party game are shown in Figure 8.

**6.4 The impact of strict supervision by government regulators**

Set$g=\{0.0,0.5,1.0\}$, the evolution process and results of the main strategies of the four-party game are shown in Figure 9.

**6.5 Impact of cost**

Suppose a drug wholesale enterprise discovers low-quality drugs and reports them. Set,and. The strategic evolution process and results of the three-party game between drug production enterprises, third-party testing agencies and government regulators are shown in Figure 10.
