# Supplementary material for: Pharmaceutical enterprises integrity supervision strategy when considering rent-seeking behavior and government reward and punishment mechanism
Source: PLoS One. 2025 May 19;20(5):e0320964. doi: 10.1371/journal.pone.0320964 (PMC12088600; doi:10.1371/journal.pone.0320964)
Supplement: Attachment — Submitted filename: Response to Reviewers.docx [file pone.0320964.s003.docx]

**Responses to Editor and** **Reviewers**

**Dear Editor Professor** **Avanti Dey and Reviewers,**

Thank you very much for giving us an opportunity to submit a revised version of the manuscript entitled “*Pharmaceutical Enterprises Integrity Supervision Strategy When Considering Rent-seeking Behavior and Government Reward and Punishment Mechanism*.” The manuscript is a revised submission (Manuscript ID: ***PONE-D-24-28268***) with new page numbers in the text, some grammar and spelling errors have also been corrected. The revised paper contains 36 pages, 10 figures and 4 tables.

We also thank the reviewers for their critical reading and valuable feedback on the manuscript. These comments provide direction for our further research. According to the reviewer's comments, relevant modifications have been made to the original manuscript, with the main changes marked in different colors. We mark the comments of the first reviewer in purple, the comments of the second reviewer in blue, the comments of the third reviewer in green, and the suggestions of the fourth reviewer in red. We also responded to each reviewer's comments listed below one by one and clearly indicated the location of the revisions. In addition, after careful consideration, we have made further improvements to the paper without changing its structure and general content, and have marked it in bold blue font.

**Responds to the editor ’s comments:**

**Reviewer #1**

**Comment #1:**

Please introduce the definition of rent-seeking behavior and its practical consequences in the introduction section.

**Response**: Thank you very much for your suggestion. As you suggested, we should introduce the definition of rent-seeking behavior and its practical consequences in the introduction part. We have added to the first paragraph of the introduction (the first paragraph on the second page). Added as: "The integrity of pharmaceutical enterprises is an embodiment of national governance capabilities and an important guarantee for the healthy development of the economy and society. The lack of integrity in the pharmaceutical field seriously affects the normal operation of the medical industry, especially when rent-seeking behaviors are involved. Rent-seeking behavior refers to the fact that enterprises or individuals obtain economic benefits through non-market means, such as bribery or interest transfer, rather than achieving profit growth through improving efficiency or innovation. In the pharmaceutical industry, drug production enterprises influence the test results of third-party testing agencies through rent-seeking behavior, which not only distorts normal medical behaviors and reduces the quality of medical services, but also may allow unqualified drugs to enter the market, seriously threatening the life safety of patients. From an ethical perspective, rent-seeking behavior places economic interests above patients' health and is a betrayal of professional spirit and social responsibility. At the same time, rent-seeking behavior leads to unfair allocation of medical resources, exacerbates excessive medical treatment and drug price increases, increases the economic burden on patients, and ultimately hinders the healthy and sustainable development of the pharmaceutical industry. Against this background, it is urgent to strengthen corporate governance to ensure that corporate behaviors comply with ethical norms and legal regulations, thereby reshaping industry trust and promoting a virtuous cycle in the pharmaceutical industry."

**Comment #2:**

In the abstract, there is one of the findings "Reducing the costs of stringent government supervision and increasing the speculative costs of rent-seeking for third-party testing agencies help prevent dishonest practices among drug production enterprises". (1) Why the impact of regulatory costs and rent-seeking costs are discussed on the dishonest practices among drug production enterprises? (2) The guidance on the decision-making of all parties should be added in the discussion section.

**Response**: (1) Thank you for your in-depth attention and professional suggestions on our research. We explore the influence of supervision costs and rent-seeking costs on the dishonest behaviors of drug production enterprises. The core purpose is to reveal and quantify how these costs play a role in the integrity supervision of pharmaceutical enterprises. The level of supervision costs directly affects the supervision intensity and efficiency of government regulators, and then determines the binding force on pharmaceutical enterprises. When the supervision costs are too high, it may weaken the supervision ability of government regulators and provide opportunities for the dishonest behaviors of drug production enterprises; conversely, reasonable and appropriate supervision costs can effectively deter illegal behaviors and promote the integrity-based operation of enterprises. Meanwhile, rent-seeking costs, that is, the risks and costs that enterprises need to bear in order to evade supervision, are directly related to whether they choose to avoid supervision through illegal means such as bribery. Higher rent-seeking costs can significantly suppress the rent-seeking motives of enterprises, forcing them to tend to abide by the law with integrity; otherwise, it may intensify unfair competition in the market and affect the health of the industry. Therefore, in-depth analysis of the specific impacts of supervision costs and rent-seeking costs on drug production enterprises not only provides theoretical support for constructing an efficient supervision system but also has important guiding significance for actual policy formulation and implementation, which is helpful to form a good situation of strict government supervision, enterprise self-discipline and social co-governance, and safeguard the healthy development of the pharmaceutical industry and the health rights and interests of the public.

(2) Thank you very much for your suggestion. As you suggested, we have revised the discussion part and provided more specific suggestions for each entity (on page 29). We added, "This paper constructs an evolutionary game model for the integrity supervision of pharmaceutical enterprises, analyzes the influence of various key factors on strategy evolution, and proposes targeted decision-making suggestions for all parties. When deeply exploring the theoretical effects of the reward and punishment mechanism, we also realize the complexity and challenges of its implementation under the existing regulatory framework.

Firstly, drug production enterprises, as the cornerstone of market integrity, their behaviors are directly related to public health and industry reputation. They should actively choose integrity-based operation and establish a complete internal monitoring and compliance system to ensure product quality and safety. Especially when facing third-party testing agencies, they need to adhere to transparent cooperation and resolutely resist rent-seeking behaviors. Under the guidance of the government's reward and punishment mechanism, enterprises should strengthen communication with regulatory departments and participate in integrity evaluations to enhance their corporate images.

Secondly, the credibility of third - party testing agencies is crucial to the healthy development of the pharmaceutical market. Strengthening internal management and ensuring the fairness and transparency of testing processes are the keys to enhancing their international competitiveness. Refusing rent-seeking behaviors not only consolidates their own market positions but also significantly enhances the willingness of enterprises to operate with integrity. Testing agencies should regard fair testing as their mission. By actively accepting supervision, improving testing levels and service qualities, they can win market trust, avoid short-term interests harming long - term reputations, and promote the healthy growth of the global pharmaceutical industry.

In addition, the strictness and fairness of government regulators are the last line of defense for maintaining market order and public health. By optimizing the reward and punishment mechanism, they can not only encourage integrity-based operation but also effectively curb rent-seeking and dishonesty. However, although theoretical analysis shows that increasing the punishment intensity has a significant effect on suppressing rent-seeking behaviors, under the existing regulatory framework, the implementation of the reward and punishment mechanism faces multiple challenges. The limited regulatory resources restrict regulatory agencies from comprehensively monitoring illegal behaviors, especially in highly specialized and information-asymmetric fields such as the pharmaceutical industry. Excessive punishment may inhibit the innovation motivation of enterprises, while too-light punishment cannot form an effective deterrence. Therefore, when designing the reward and punishment mechanism, the government needs to balance the intensity of punishment within the legal framework, and at the same time consider industry characteristics, enterprise scales, and the nature of illegal behaviors to ensure the fairness and rationality of the mechanism. Specifically, the government should start from two aspects. Firstly, clarify and increase the economic punishment intensity for rent-seeking behaviors of pharmaceutical enterprises to make it exceed a certain threshold. By significantly increasing the amount of fines to exceed the potential benefits brought by rent-seeking behaviors, the illegal cost of enterprises can be effectively increased. For serious dishonest behaviors, the economic punishment should be sufficient to weaken the competitiveness of enterprises in the market and may even lead to enterprise bankruptcy liquidation to form a strong deterrent effect. Secondly, the government should consider implementing criminal punishments. In key areas involving public health and safety, the pursuit of criminal liability is crucial. For behaviors of serious rent-seeking or causing major damage consequences, the government can pursue the criminal liability of relevant responsible persons in accordance with the law, implement punitive detention, deprive of business qualifications and other measures, thereby enhancing the deterrence of the law. In addition, in order to effectively curb rent-seeking, the blacklist system should be strengthened, a public supervision and reporting mechanism should be established, and multi-department joint law enforcement and other measures should be considered. Although these policy tools will increase regulatory costs in the short term, in the long run, by improving regulatory effects and reducing illegal behaviors, the overall regulatory cost can be reduced, and at the same time, market confidence and stability can be enhanced, thereby reducing the costs of supervision and law enforcement.

Finally, drug wholesale enterprises, as the bridge connecting production and consumption, are crucial in maintaining market order through the reporting mechanism. Enterprises should establish and improve internal reporting and feedback mechanisms to ensure that problem products are reported quickly. The government should provide convenient reporting channels for wholesale enterprises, protect the rights and interests of whistleblowers, and give appropriate rewards to stimulate their enthusiasm for participating in supervision.

In conclusion, the government, enterprises, and third-party testing agencies need to flexibly adjust strategies according to their respective national conditions to jointly promote the construction of the integrity supervision system in the pharmaceutical industry and promote the healthy development of the global pharmaceutical market and the protection of public health. However, it is worth noting that the model construction and analysis in this study are mainly based on the game theory framework, assuming that all participants make decisions based on bounded rational behavior. This assumption simplifies the complexity of the real world to a certain extent. Therefore, although the model can reveal the effect of the reward and punishment mechanism under ideal conditions, when applied to actual scenarios, the uncertainty brought by irrational behaviors and the possibility of deviation from predicted results need to be considered. To make up for this limitation, future research can try to introduce the perspective of behavioral economics, incorporate irrational behaviors into model consideration, in order to more comprehensively reflect the decision-making process in the real world and thus improve the prediction accuracy and application value of the model."

**Comment #3:**

Please explain why evolutionary game theory is used to solve the problem in this paper.

**Response**: Thank you for your attention and guidance on our research. We choose game theory as an analytical tool mainly because the issue of integrity supervision of pharmaceutical enterprises involves interactions and strategy selections among multiple entities, which is exactly the core research area of game theory. In the context of integrity supervision in the pharmaceutical industry, the relationships among drug production enterprises, third-party testing agencies, government regulators, and drug wholesale enterprises are complex. Each party makes decisions based on its own interests, and these decisions influence each other, forming a dynamic strategic game. By constructing an evolutionary game model, we can systematically analyze the strategy selection processes of each entity, understand the influence of the reward and punishment mechanism on strategy evolution, and then explore the optimal strategy combination, which provides a theoretical basis for improving supervision efficiency and promoting integrity-based operation. In addition, the game theory analysis also helps us quantify the influence of each decision variable on the strategy selections of different entities. Through simulation analysis, the effectiveness of the theoretical model is verified, providing specific guidance for policy formulation and practical application. Therefore, we have made a supplement in the Problem Description part of 3.1 (the last paragraph on page six). The supplementary content is: "Evolutionary game theory, as a powerful analytical tool, has the core advantage of being able to depict and predict the dynamic strategic interactions among multiple agents. It is especially suitable for research scenarios involving complex decision-making environments such as integrity supervision of pharmaceutical enterprises. By using evolutionary game theory, we can deeply analyze the decision-making process of each participating party when facing the reward and punishment mechanism and reveal the mechanism of action of the reward and punishment mechanism on strategy evolution. Therefore, evolutionary game theory has strong applicability and effectiveness in solving the problem of integrity supervision of pharmaceutical enterprises. "

**Comment #4:**

There are some grammar errors.

**Response**: Sincerely thank you for the valuable comments you put forward on our paper. The grammar mistakes you pointed out are indeed the areas that we need to attach importance to and correct. We deeply understand the importance of accurate expression for the quality of academic papers. We have now carried out a detailed grammar check and correction on the full text to improve the readability and professionalism of the paper.

**Comment #5:**

The content of the manuscript is too long. The proofs can be appropriately simplified.

**Response**: Thank you very much for your valuable suggestions. We understand that the conciseness of the paper content is crucial for clearly conveying the research focus. We have followed your advice to appropriately simplify the proof process, ensuring that the paper content is more concise while still maintaining the integrity and rigor of the argument.

**Reviewer #2**

**Comment #1:**

The introductory section provides an overview of pharmaceutical corporate integrity and its importance, but could be further enhanced with a critical analysis of the existing literature, clearly identifying the shortcomings of existing research and the innovations of this study.

**Response**: Thank you for your valuable suggestions. According to your suggestions, we have revised the 2 Literature review part (the last two paragraphs on page 5), strengthened the critical analysis of the existing literature, and highlighted the research contributions of this study. The specific revision is: "All in all, the existing literature has made great research contributions to promoting the integrity operation of pharmaceutical enterprises, but there are still deficiencies. Firstly, in the research on the integrity issues of pharmaceutical enterprises [1-7], the existing literature seldom discusses from the perspective of multi-agent collaborative supervision, and the research on multi-agent behavior games and strategy selections is relatively blank. Secondly, the research on government integrity supervision is very rich [8-21], but it mostly focuses on the design and optimization of the supervision mechanism, while the dynamic effect analysis of the reward and punishment mechanism is still insufficient. Finally, although the existing research has emphasized the supervisory role of third-party agencies [22-27], there are still gaps in the analysis of how they make strategy selections under the reward and punishment mechanism and how they interact with the whistle - blowing behavior of drug wholesale enterprises.

The research contributions of this paper are as follows: Firstly, considering multi-agent collaborative supervision, an evolutionary game model participated by drug production enterprises, third-party testing agencies, government regulators and drug wholesale enterprises is constructed. Secondly, considering the influence of the behaviors of third-party testing agencies and drug wholesale enterprises on the strategy selections of other participating agents under the reward and punishment mechanism, the stable equilibrium points of strategy choices under different conditions are solved. Finally, the influence of each decision variable is analyzed, and Matlab 2022b is used for simulation analysis to verify the effectiveness of the model, and countermeasures and suggestions for the integrity supervision of pharmaceutical enterprises are put forward."

**Comment #2:**

Subsection 2.1 refers to pharmaceutical corporate integrity issues, but does not elaborate on how these issues specifically affect public health and industry development.

**Response**: Thank you for your valuable suggestions. We have re-organized the 2.1 Integrity Issues of Pharmaceutical Enterprises part and elaborated as much as possible on how the integrity issues of pharmaceutical enterprises specifically affect public health and industry development (the last paragraph on page 3). After the revision, this part is as follows: "The integrity issues of pharmaceutical enterprises involve complex interest relationships and multiple subjects. The untrustworthy behaviors of enterprises will directly affect the quality and safety of drugs, and further endanger public health. For instance, untrustworthy enterprises may utilize inferior or unqualified raw materials to cut down production costs, thereby directly threatening the lives and health of patients (Xu Y., et al, 2023; Rong J., et al, 2020) [1][2]. Furthermore, the lack of integrity in pharmaceutical enterprises may lead to a decline in social trust, exacerbate the unfairness of pharmaceutical resources, and ultimately harm social stability and national security (Helmut B., et al, 2024) [3]. However, restricted by production costs, enterprise scale and high investment in drug research and development, opportunistic behaviors and rent - seeking phenomena in enterprises occur frequently (Busby J.S., 2019) [4]. Existing research also indicates that in the supply chains of many countries, unqualified or counterfeit drugs account for a certain proportion, which undermines market fairness and consumer rights (Baptiste B., et al, 2022) [5]. In particular, the COVID - 19 pandemic in 2019 exposed a large number of untrustworthy problems, making the government pay more attention to the integrity issues of pharmaceutical enterprises (Ranjana P., et al, 2023; Bao Y., et al, 2020) [6][7]. "

**Comment #3:**

Subsection 2.2 discusses government regulation of integrity and suggests adding some analyses on cases of regulatory failures to enhance the persuasiveness of the argument.

**Response**: Thank you for your valuable suggestions. We agree with your view that introducing case analysis of supervision failures in the 2.2 Government Integrity Supervision part can strengthen the practicality of the research and enhance the persuasiveness of the argument. We have added case content (in the second paragraph on page 4) in the part. The specific added content is: "Although government regulation is regarded as an effective way to ensure the honest operation of pharmaceutical enterprises (Magdalena B., et al, 2021)[8], cases of regulatory failures, such as the vaccine fraud incident of Changchun Changsheng Bio - tech Co., Ltd. in China in 2018, rent-seeking incidents in Pfizer and Johnson & Johnson, have exposed the blind spots of government regulation (Flynn M., 2018)[9]. These incidents highlight the issues of information transparency and enforcement in government regulation and emphasize the importance of improving the regulatory system."

**Comment #4:**

The model assumptions (H1-H6) provide the basis for model construction, but there is a need to ensure that these assumptions are well grounded and supported by data in the real world.

**Response:** Thank you very much for your attention to this article and your valuable suggestions. Your professional feedback is of great significance for us to improve the research. When constructing the model assumptions, we deeply integrated real - data and industry insights to ensure the rigor and practicality of the assumptions. Referring to official materials such as "2022 Annual Statistics on Drug Supervision and Administration", "2023 Department Budget of the National Medical Products Administration", "FDA Annual Performance and Financial Report", "Overview of Health Economic Data in the UK" and "World Health Statistics", combined with literature review, industry status analysis and expert interviews, we carefully calibrated the model parameters, aiming to reflect the overall picture of the industry. Although the secrecy of rent-seeking behavior and the challenges of data acquisition limit the accuracy of some parameters, we still continuously verify and improve through simulation analysis and continuous research, striving to make the model assumptions be both rooted in reality and forward-looking, so as to provide solid support for the research. Therefore, we added a detailed explanation in 3.2 Model Assumptions (the second paragraph on page 7). The supplementary content is: "In view of the secretive nature of rent-seeking behavior and the sensitivity of relevant data, when setting the model parameters in this study, official-released data was widely referenced, such as "2022 Annual Pharmaceutical Supervision and Administration Statistics Data", "2023 Department Budget of the National Medical Products Administration", "FDA Annual Performance and Financial Report", "Overview of Health Economic Data in the UK" and "World Health Statistics", to ensure the rationality of key parameters. At the same time, based on literature review and comprehensive analysis of the industry situation, supplemented by field research and interviews with industry experts, we have formed a preliminary estimate of the model parameters and made the following hypotheses."

**Comment #5:**

In the model construction section, it is recommended that more details be provided, such as the selection of variables and the method of determining parameters.

**Response:** Thank you very much for your attention to this paper and your valuable comments. Your professional feedback is of great significance for us to improve the research. In the model assumption part (the second paragraph on page 7), we explained the method of variable selection and parameter determination. We added the following content: "In view of the secretive nature of rent-seeking behavior and the sensitivity of relevant data, when setting the model parameters in this study, official-released data was widely referenced, such as "2022 Annual Pharmaceutical Supervision and Administration Statistics Data", "2023 Department Budget of the National Medical Products Administration", "FDA Annual Performance and Financial Report", "Overview of Health Economic Data in the UK" and "World Health Statistics", to ensure the rationality of key parameters. At the same time, based on literature review and comprehensive analysis of the industry situation, supplemented by field research and interviews with industry experts, we have formed a preliminary estimate of the model parameters and made the following hypotheses."

**Comment #6:**

the section provides mathematical analyses of the stability of the strategies of different participants. It is recommended that visual explanations of these mathematical models be added to aid understanding by non-specialist readers. In addition, the authors should include in the analysis a sensitivity analysis for different parameter variations. It is recommended to refer to the following references. Tripartite evolutionary game analysis and simulation research on zero-carbon production supervision of marine ranching against a carbon-neutral background；A tripartite evolutionary game study of low-carbon innovation system from the perspective of dynamic subsidies and taxes；Research on the tripartite evolutionary game of public participation in the facility location of hazardous materials logistics from the perspective of NIMBY events.

**Response:** In response to the suggestions put forward by the reviewers, we deeply feel their constructiveness and importance, and hereby extend our sincere gratitude. In order to improve the convenience for non-professional readers to understand the model stability and strategy evolution, we have carefully added the evolution phase diagrams of strategy choices of all parties with reference to the literature you recommended, which intuitively show the dynamic changes of strategies under parameter changes and enhance the readability of the article and the intuitiveness of the analysis.

In the simulation analysis part, we have made meticulous adjustments. We not only more intuitively show the influence of the changes in the reward and punishment amounts of government regulators for drug wholesale enterprises, and the fine amounts for drug production enterprises and third-party testing agencies on strategy evolution, but also deeply discuss the dynamic influences of the reporting behavior of drug wholesale enterprises, the strict supervision cost of government regulators, and cost factors on strategy selection. Through simulation analysis, we can intuitively see how various key elements affect the strategy selection of different entities. This is essentially in line with the goal of sensitivity analysis, that is, to evaluate the impact of parameter changes on model output. However, simulation analysis focuses more on the visual presentation of strategy evolution paths and dynamic equilibrium points, providing direct empirical support for strategy optimization. By simulating the strategy evolution processes under different parameters, simulation analysis not only verifies theoretical assumptions, but also reveals how policy - makers can guide market behavior and optimize supervision strategies by adjusting the reward - punishment mechanism. Its results provide a strong basis for policy-making and show the value of the model in practical applications.

We believe that through the above-mentioned improvements, the depth and breadth of the article's analysis have been significantly enhanced, and the mechanism of action of the government's reward and punishment mechanism in the integrity supervision of pharmaceutical enterprises has been more comprehensively demonstrated. Thank you again for your valuable suggestions. We have incorporated your suggestions into the article. The phase diagrams are shown as follows.

**Figure 2 Phase diagram of drug production enterprises strategic choices**

**Figure 3 Phase diagram of third-party testing agencies strategic choices**

**Figure 4 Phase diagram of government regulators strategic choices**

**Figure 5 Phase diagram of drug wholesale enterprises strategic choices**

**Comment #7:**

The simulation analyses were partially conducted using Matlab 2022b, but no detailed description of the code or the models was provided. To increase transparency and reproducibility, it is recommended that the simulation code or at least key code segments be provided.

**Response:** We sincerely thank the reviewers for their valuable suggestions and deeply understand the value of providing simulation code for enhancing the transparency and reproducibility of the research. In view of this, we will attach the key parts of the simulation code for Matlab 2022b to ensure that peers and readers can easily obtain and reproduce our research results.

The key code segments for the two-dimensional plan view are: "clc;clear;

set(0,'defaultfigurecolor','w')

[t,y]=ode45(@(t,y) lunwen2pingmian(t,y,Ch,Fp,Cl,Cp,W,Ft,Cs,Bt,Fw,C1,Dg,Mw,C2,Cr,Rl,Fg),[0,200],[0.5,0.5,0.5,0.5]);

points=1:1:length(t);

plot(t,y(:,1),'g-','linewidth',3,'markersize',3,'markerfacecolor','r','markerindices',points);

hold on

plot(t,y(:,2),'m-.','linewidth',3);

hold on

plot(t,y(:,3),'b-.','linewidth',3);

hold on

plot(t,y(:,4),'r-.','linewidth',3);

hold on

set(gca,'XTick',[0:3:30],'YTick',[0.0:0.2:1.0])

axis([0 30 0 1])

xlabel('Time','interpreter','latex');

ylabel('Strategic choice');

legend('drug production enterprises','third-party testing agencies','government regulators','drug wholesale enterprises');"

The key code segments for the three-dimensional graph are " for i=0.1:0.2:1

for j=0.1:0.2:1

for k=0.1:0.2:1

[t,y]=ode45(@(t,y)lunwen2sanfang(t,y,Ch,Fp,Cl,Cp,W,Ft,Cs,Bt,Fw,C1,Dg,Mw,C2,Cr,Rl,Fg),[0 500],[i j k]);

figure(1)

grid on

plot3(y(:,1),y(:,3),y(:,2),'linewidth',1);

set(gca,'XTick',[0:0.2:1],'YTick',[0:0.2:1],'ZTick',[0:0.2:1])

hold on

axis([0 1 0 1 0 1])

view([45 10])

end

end

end

xlabel('x','Rotation',0);

ylabel('y','Rotation',0);

zlabel('g','Rotation',360,'position',[-0.23 0.25 0.4]);"

**Comment #8:**

The graphs (Figure 6 to Figure 10) provide a visual representation of the simulation results, but there is a need to ensure that each graph has a detailed legend and explanation.

**Response:** We are very grateful to the peer reviewers for their valuable suggestions on the article. Indeed, clear and detailed explanations of figures and tables are crucial for understanding the research results. Regarding Figure 6 to Figure 10 you mentioned, we have supplemented detailed legends and explanations for each figure. This will include, but not be limited to, the definitions of specific parameters, the meanings of variables, and the specific situations represented by each data point or trend line, ensuring that readers can fully understand the figure information at a glance without additional inquiries. In addition to the detailed analysis of figures, we have also supplemented figure legends (on page 35), and the specific content is:

"**Figure 1** Four-party game relationship

Figure 1 is a structural diagram of the relationship between drug production enterprises, third-party testing agencies, government regulators and drug wholesale enterprises that introduce government reward and punishment mechanisms under rent-seeking behavior.

**Figure 2** Phase diagram of drug production enterprises strategic choices

Figure 2 is the phase diagram that shows the evolutionary trend of drug production enterprises 's strategy obtained by calculating the response function of the probability of drug production enterprises choosing the "integrity management" strategy.

**Figure 3** Phase diagram of third-party testing agencies strategic choices

Figure 3 is the phase diagram that shows the evolutionary trend of third-party testing agencies' strategy obtained by calculating the response function of the probability of third-party testing agencies choosing the "accept rent-seeking" strategy.

**Figure 4** Phase diagram of government regulators strategic choices

Figure 4 is the phase diagram that shows the evolutionary trend of government regulators' strategy obtained by calculating the response function of the probability of government regulators choosing the "strict supervision" strategy.

**Figure 5** Phase diagram of drug wholesale enterprises strategic choices

Figure 5 is the phase diagram that shows the evolutionary trend of drug wholesale enterprises' strategy obtained by calculating the response function of the probability of drug wholesale enterprises choosing the "reporting" strategy.

**Figure 6** The impact of fines and incentives on drug wholesale enterprises

Figure 6 is the simulation diagram that shows the influence of the reward amount brought by drug wholesale enterprises reporting and the fine amount brought by drug wholesale enterprises not reporting on the strategic choices of drug production enterprises, third-party testing agencies, government regulators, and drug wholesale enterprises. (A) When the situation,. (B) When the situation,. (C) When the situation,.

**Figure 7** The impact of fines on drug production enterprises and third-party testing agencies

Figure 7 is the simulation diagram that shows the influence of the fine amount brought by drug production enterprises dishonest management and third-party testing agencies accept rent-seeking on the strategic choices of drug production enterprises, third-party testing agencies, government regulators, and drug wholesale enterprises. (A) When the situation,. (B) When the situation,. (C) When the situation,.

**Figure 8** The impact of reporting by drug wholesale enterprises

Figure 8 is the simulation diagram that shows the influence of the probability of reporting by drug wholesale enterprises on the strategic choices of drug production enterprises, third-party testing agencies, and government regulators. (A) When the situation$z=0.0$. (B) When the situation$z=0.5$. (C) When the situation$z=1.0$.

**Figure 9** The impact of strict supervision by government regulators

Figure 9 is the simulation diagram that shows the influence of the probability of strict supervision by government regulators on the strategic choices of drug production enterprises, third-party testing agencies, and drug wholesale enterprises. (A) When the situation$g=0.0$. (B) When the situation$g=0.5$. (C) When the situation$g=1.0$.

**Figure 10** Impact of cost

Figure 10 is the simulation diagram that shows the influence of various costs on the strategic choices of drug production enterprises, third-party testing agencies, and government regulators. (A) When the situation,,. (B) When the situation,,. (C) When the situation,,."

We promise that in the subsequent versions, these supplementary explanations will be presented in the most intuitive and clear way to improve the overall readability and professionalism of the article. Thank you for your guidance. Your suggestions will greatly help us improve the quality of the article."

**Comment #9:**

The discussion section provides some policy recommendations, but it is recommended that the applicability and feasibility of these recommendations in different countries and regions be explored in more depth.

**Response:** Thank you for your valuable comments on this article. We attach great importance to your suggestion of conducting in-depth exploration on the applicability and feasibility of policy recommendations in the discussion part in different countries and regions. Indeed, considering the universal goal of this research, we focus on extracting the general theoretical framework of integrity supervision of pharmaceutical enterprises under rent-seeking behavior and the effectiveness of the reward and punishment mechanism, aiming to provide a model for policymakers around the world to think about and draw lessons from. We understand that differences in laws and regulations, market maturity, cultural backgrounds, etc. among different countries and regions will indeed affect the direct application of policy recommendations. In this research, through extensive reference to international data and literature and in combination with industry expert interviews, we strive to ensure the rationality and universal applicability of model parameter settings. However, we admit that when specific policy recommendations are implemented in different countries and regions, they do need to be appropriately adjusted and localized according to local specific situations. In response to your suggestion, we plan to conduct a more in-depth exploration of the applicability and feasibility of policy recommendations through further cooperation with policymakers, scholars, and industry experts in various countries in future research. This will include case studies, empirical analysis, and policy simulations for different countries and regions to more specifically evaluate and optimize our recommendations. We believe that through such efforts, more accurate and effective policy guidance for integrity supervision in the global pharmaceutical industry can be provided. Thank you again for your valuable comments. Your feedback will greatly promote the deepening and expansion of our research. We look forward to being able to better meet your suggestions in subsequent research and make greater contributions to the theory and practice in the field of global pharmaceutical supervision.

**Comment #10:**

The Recommendations Discussion section includes a discussion of the limitations of the model and how these limitations may affect the findings and recommendations.

**Response:** Thank you for your suggestions. According to your suggestions, in the "7 Discussions" part, we added the consideration of "the limitations of the model and how these limitations may affect the findings and recommendations" (the third paragraph on page 31). The supplementary content is: "In conclusion, the government, enterprises, and third-party testing agencies need to flexibly adjust strategies according to their respective national conditions to jointly promote the construction of the integrity supervision system in the pharmaceutical industry and promote the healthy development of the global pharmaceutical market and the protection of public health. However, it is worth noting that the model construction and analysis in this study are mainly based on the game theory framework, assuming that all participants make decisions based on bounded rational behavior. This assumption simplifies the complexity of the real world to a certain extent. Therefore, although the model can reveal the effect of the reward and punishment mechanism under ideal conditions, when applied to actual scenarios, the uncertainty brought by irrational behaviors and the possibility of deviation from predicted results need to be considered. To make up for this limitation, future research can try to introduce the perspective of behavioral economics, incorporate irrational behaviors into model consideration, in order to more comprehensively reflect the decision-making process in the real world and thus improve the prediction accuracy and application value of the model."

**Comment #11:**

The conclusion section succinctly summarises the key findings of the study. It is recommended that the authors consider adding specific recommendations for future research directions and how these findings can be applied in practice.

**Response:** Thank you for your suggestions. According to your suggestions, we have revised the "8 Conclusions" part (the last paragraph on page 32). The supplementary content is: "Considering the strategy choices of drug production enterprises, third-party testing agencies, government regulators, and drug wholesale enterprises, this paper constructs a four-party evolutionary game model. This paper studies the role of the government's reward and punishment mechanism in the integrity supervision of pharmaceutical enterprises. We realize that the reputation mechanism of social media, with its unique reputation punishment and reward functions, also plays an indispensable role in the integrity supervision of pharmaceutical enterprises. Therefore, future research should deeply explore the quantitative influence of the social media reputation mechanism, including the specific correlation between public feedback on social media and corporate behaviors, and how to further enhance the integrity awareness of pharmaceutical enterprises by optimizing social media supervision policies, so as to construct a more complete and effective integrity supervision system. Through empirical analysis of the role of the social media reputation mechanism in the integrity supervision of pharmaceutical enterprises, data support can be provided for policy-makers, guiding the government, enterprises, and third-party agencies on how to use social media to improve supervision efficiency in practical operations, and jointly create a more integrity-based, transparent, and healthy pharmaceutical market environment."

**Reviewer #3**

**Comment #1:**

Could you add parameters in Figure 1? It is difficult to understand the relationship among parameters from the current manuscript.

**Response:** Thank you for your suggestion. We have added the probability parameters of each party's strategy choice in Figure 1 to better understand the game relationship among all parties (on page 7). The game relationship diagram after supplementation is shown as follows.

**Figure 1 Four-party game relationship**

**Comment #2:**

The information in Section 3.1 is hard to understand. A table related to the hypotheses would be helpful.

**Response:** Thank you for your suggestions. We have added Table 1 "Description of relevant parameters" in section 3.1, so as to better understand the information in 3.1 (on page 9).

**Table 1 Description of relevant parameters**

| **symbol** | **describe** | **symbol** | **describe** |
| --- | --- | --- | --- |
| $g$ | The probability that the government will choose strict regulation |  | The amount of fines imposed by superior governments on government regulators |
| $x$ | The probability of drug production enterprises choosing to operate with integrity |  | Speculation costs of drug production enterprises |
| $y$ | The probability that a third-party testing agency chooses to accept rent seeking |  | Fines for drug wholesale enterprises |
| $z$ | The probability of drug wholesale enterprises choosing to report | $R$ | Profit of drug wholesale enterprises |
|  | The cost of producing low-quality drugs |  | Fines for third-party testing agencies |
|  | The cost of producing high-quality drugs |  | Incentive amount for drug wholesale enterprises |
|  | Rent-seeking cost |  | Decline in government credibility |
|  | Testing income |  | The cost of strict government regulation |
|  | The speculative costs of accepting rent-seeking |  | The cost of loose government regulation |
| $W$ | Drug wholesale prices | $L$ | Reputational loss |
|  | Testing cost |  | Fines for drug production enterprises |
| $I$ | Reputation value premium | $S$ | Increase government credibility |
|  | Amount of compensation |  | Reporting cost |

**Comment #3:**

Is the model itself novel? Please clarify it.

**Response:** Thank you very much for your attention to this article and your valuable comments. Your professional feedback is of great significance for us to improve the research. Regarding the novelty of the model you mentioned, we will explain it in detail here:

Firstly, the four-party evolutionary game model we constructed incorporates drug production enterprises, third-party testing agencies, government regulators and drug wholesale enterprises into the same framework for analysis, which is a relatively rare integrated perspective in the existing literature. Through this model, we not only explored the strategic interactions among various subjects, but also analyzed in-depth the impact of the government's reward and punishment mechanism, the credibility of third-party testing agencies, and the reporting behavior of drug wholesale enterprises on the integrity supervision system of the entire pharmaceutical industry, thus providing a new theoretical perspective for the integrity supervision of pharmaceutical enterprises.

Secondly, in the research, Lyapunov's first method is used to analyze the stability of strategy combinations, and Matlab 2022b is used for simulation analysis. This not only verifies the effectiveness of the model, but also intuitively shows the influence of various decision variables on the strategy selection of different subjects. The application of this methodology provides a new empirical analysis tool for the research on the integrity supervision of pharmaceutical enterprises.

Thirdly, this article not only focuses on theoretical modeling, but also puts forward specific suggestions for practical applications, including the optimization of the government's reward - punishment mechanism, the standardized management of third-party testing agencies, and the improvement of the reporting mechanism of drug wholesale enterprises, aiming to provide practical suggestions for policy-makers and promote the healthy development of the pharmaceutical industry.

In conclusion, this article reflects innovation in model construction, analysis methods, and practical application suggestions. We believe that through the research in this article, new insights and inspirations can be brought to the theory and practice in the field of integrity supervision of pharmaceutical enterprises.

Thank you again for your attention and guidance on this article.

**Comment #4:**

Which assumption is the most critical for your results? Also, which parameter is the most sensitive for your results? Please clarify them quantitatively.

**Response:** Thank you for your suggestions. In our research, indeed, several key assumptions and parameters have a significant impact on the results.

Firstly, the most crucial assumption is that the loose supervision of government regulators and the rent-seeking acceptance of third-party testing agencies are both unfavorable to integrity-based operations. We analyzed the cost parameters. When the rent-seeking cost of drug production enterprises is assumed to be 2.9, the extra cost of strict government supervision is 13, and the reporting cost of drug wholesale enterprises is 4, the probability of strict government supervision is 0, the probability of third-party testing agencies rejecting rent-seeking is 1, and the probability of drug production enterprises operating with integrity is 0. When the rent-seeking cost of drug production enterprises is assumed to be 6.9, the extra cost of strict government supervision is 3, and the reporting cost of drug wholesale enterprises is 6, the probability of strict government supervision is 1, the probability of third-party testing agencies rejecting rent-seeking is 0, and the probability of drug production enterprises operating with integrity is 0. This result confirms the rationality of the assumption.

Secondly, regarding parameter sensitivity, the fine amount when drug wholesale enterprises do not report and the reward amount when they report are sensitive parameters. When the fine amount and the reward amount are 0, the probability of drug wholesale enterprises choosing to report is 0, and the probability of third - party testing agencies rejecting rent-seeking is 0.3. When the fine amount and the reward amount increase to 7, the probability of drug wholesale enterprises choosing to report is 1, and the probability of third-party testing agencies rejecting rent-seeking is 0.9. This indicates that optimizing the reporting mechanism is an effective strategy for enhancing the market integrity level.

In addition, the fine amounts imposed by government regulators on drug production enterprises and third-party testing agencies are also very sensitive parameters. When the fine amount for the dishonest operation of drug production enterprises and the fine amount for the rent-seeking acceptance of third-party testing agencies are 0, the probability of drug production enterprises operating with integrity is 0, and the probability of third-party testing agencies accepting rent-seeking is 1. When the fine amounts for the dishonest operation of drug production enterprises and the rent - seeking acceptance of third-party testing agencies increase to 10, the probability of drug production enterprises operating with integrity is approximately 0.6, and the probability of third-party testing agencies accepting rent-seeking is 0. This change highlights the direct impact of the fine amounts.

In conclusion, through carefully setting key assumptions and parameters and combining with simulation analysis, our research effectively verifies the role of the reward and punishment mechanism in promoting the integrity supervision of pharmaceutical enterprises.

**Comment #5:**

How do you manage uncertainties of your calculation results? There should be many uncertainties in your model.

**Response:** We sincerely thank you for your in-depth review and valuable suggestions on this article. We deeply agree with the issues you raised and will explain them in detail here. In the process of constructing and analyzing the evolutionary game model, we are fully aware of the differences between the model parameter settings and the real world, as well as the uncertainties that such differences may introduce. In response to this, we have taken the following measures to manage the uncertainties.

Firstly, we pay attention to the rigor of parameter settings. We have referred to official materials such as "Annual Data of Drug Supervision and Administration Statistics in 2022", "Department Budget of the National Medical Products Administration in 2023", "FDA Annual Performance and Financial Report", "Overview of Health Economic Data in the UK" and "World Health Statistics" to conduct detailed data research and settings for the key parameters in the model. This ensures the rationality of the basic parameters of the model and reduces the uncertainties caused by improper parameter settings.

Secondly, we focus on the rationality of model assumptions. In the model construction stage, based on the existing literature and the current situation of the industry, we carefully consider the model assumptions to ensure that they are in line with the actual operation logic of the pharmaceutical industry. At the same time, we have in-depth discussions on the scope of application and potential limitations of each assumption to enhance the applicability and predictive ability of the model.

Finally, we conduct simulation analysis. Through the simulation analysis carried out by Matlab 2022b, we not only verify the stability of the model, but also observe the evolution trend of strategy combinations through parameter changes. The simulation analysis itself includes an indirect examination of the sensitivity of model parameters. By observing how minor parameter adjustments affect strategy selection, we indirectly assess the robustness of the model results.

We recognize that by introducing specific cases, the stability and practicality of the model can be further verified. Therefore, in the follow-up research, we plan to collect more empirical data and introduce case studies to quantify uncertainties more scientifically and improve the accuracy and reliability of model predictions. Through the above measures, we strive to manage uncertainties to the greatest extent within the existing research framework and ensure that the research results have certain theoretical and practical guiding values in the field of integrity supervision of pharmaceutical enterprises.

We are very grateful for your professional guidance, and your suggestions will have a profound impact on our follow-up research.

**Comment #6:**

What is the key message of Figure 10? The review believes the figure is the most important result in the manuscript, so please clarify the message.

**Response:** Thank you for your detailed review and valuable comments on this article. Regarding Figure 10, it is indeed one of the core results of this research, and we are very glad to further interpret its key information for you. Figure 10 reveals the process of strategy evolution and its stable states among drug production enterprises, third-party testing agencies and government regulators in the case where drug wholesale enterprises discover low-quality drugs and report them. The strategy stable points (0, 0, 0) and (0, 1, 1) shown in the figure correspond to different supervision and market behavior patterns respectively. Specifically, when the extra cost of strict government supervision is high, while the rent-seeking cost of drug production enterprises and the reporting cost of drug wholesale enterprises are low, the system tends to the strategy stable point (0, 0, 0). At this time, government regulators may choose loose supervision to reduce costs, resulting in drug production enterprises being more likely to choose dishonest operations, and third - party testing agencies tend to reject rent-seeking behavior. As the extra cost of strict government supervision decreases, and the rent-seeking cost of drug production enterprises and the reporting cost of drug wholesale enterprises increase, the system gradually evolves from the unstable state of (0, 0, 0) to another stable point (0, 1, 1). In this state, government regulators implement strict supervision, while drug production enterprises and third-party testing agencies choose dishonest operations and accept rent - seeking behavior respectively.

The key information of Figure 10 lies in that it emphasizes the important regulatory roles of the strict supervision cost of government regulators, the rent-seeking cost of drug production enterprises and the reporting cost of drug wholesale enterprises on market behavior. And it is concluded that the loose supervision of government regulators and the acceptance of rent-seeking by third-party testing agencies are both unfavorable to the integrity-based operations of drug production enterprises. Therefore, by reasonably adjusting these costs, the integrity-based operations of drug production enterprises can be effectively promoted, the fairness of third-party testing agencies can be ensured, and the efficiency of government supervision can be achieved.

We believe that Figure 10 not only verifies the effectiveness of the model, but also provides empirical evidence for policy-makers, guiding them on how to adjust supervision policies and market mechanisms to promote the healthy and integrity-based development of the pharmaceutical industry. Thank you again for your attention and guidance.

**Reviewer #4**

**Comment #1:**

The paper contributes to pharmaceutical integrity through a strong theoretical model but needs improvement in practical applicability, empirical validation, and real-world complexity.

**Response:** We sincerely thank the reviewers for their valuable comments. Based on your suggestions, we added a new section 3.1 "Problem Description" in the "3 Model Hypotheses and Construction" part. This section is closely related to practice. Through specific scenario discussions, it not only enhances the practical guiding significance of the model but also provides policy-makers with clearer decision-making bases. We believe that this improvement not only deepens the understanding of the integrity supervision mechanism of pharmaceutical enterprises but also further verifies the applicability of the model in complex real-world environments, laying a solid foundation for future research and policy implementation (the second paragraph on page 6). The specific content is "3.1 Problem Description. In the context of globalization, the pharmaceutical industry is facing increasingly complex regulatory challenges. In particular, rent-seeking behavior erodes the integrity supervision system, threatening public health and market fairness. How to promote the integrity of pharmaceutical enterprises and build a fair and transparent market environment through effective reward and punishment mechanisms has become a key issue that urgently needs to be addressed. In response to this real-world scenario, this paper constructs an evolutionary game model involving drug production enterprises, third-party testing agencies, government regulators and drug wholesale enterprises. Through model analysis, we aim to gain a deeper understanding of the strategy choice and evolution process of each participating party under the reward and punishment mechanism, and explore how to optimize the reward and punishment mechanism to improve the efficiency of integrity supervision of pharmaceutical enterprises. This paper will provide policy makers with decision-making bases based on real market behaviors, guiding the government, enterprises and third-party agencies on how to formulate and implement more effective reward and punishment policies in specific situations, in order to promote integrity operation, maintain market order, safeguard public health, and build a harmonious pharmaceutical market environment. Therefore, the construction and analysis of this model will directly benefit from in-depth exploration of specific real-world scenarios, providing policy makers with clearer and more practical guiding directions."

In addition, this research aims to construct a theoretical framework to explore the role of the government's reward and punishment mechanism in the integrity supervision of pharmaceutical enterprises. Given the limitations of current research, we plan to strengthen the empirical basis of the model in subsequent research by introducing more case studies, empirical data analysis, and industry surveys. At the same time, we also consider cooperating with industry experts to obtain more direct industry data and insights and further verify the fit between model predictions and industry practices. Here, we sincerely ask the reviewers to understand that, given the nature and conditional limitations of current research, we are temporarily unable to conduct a comprehensive empirical verification of the model. We promise that in future research, we will focus on strengthening the support of empirical data to enhance the practicality and influence of the research. We are very grateful for the in-depth feedback you provided on this article, which will greatly promote our future research.

**Comment #2:**

The discussion on enforcing theoretical findings like increased penalties for rent-seeking is insufficient, with little exploration of challenges in applying the reward and punishment mechanisms in existing regulatory frameworks.

**Response:** Thank you for your suggestions. We have made supplementary modifications in the "7 Discussions" part (on page 29). We added " This paper constructs an evolutionary game model for the integrity supervision of pharmaceutical enterprises, analyzes the influence of various key factors on strategy evolution, and proposes targeted decision-making suggestions for all parties. When deeply exploring the theoretical effects of the reward and punishment mechanism, we also realize the complexity and challenges of its implementation under the existing regulatory framework.

Firstly, drug production enterprises, as the cornerstone of market integrity, their behaviors are directly related to public health and industry reputation. They should actively choose integrity-based operation and establish a complete internal monitoring and compliance system to ensure product quality and safety. Especially when facing third-party testing agencies, they need to adhere to transparent cooperation and resolutely resist rent-seeking behaviors. Under the guidance of the government's reward and punishment mechanism, enterprises should strengthen communication with regulatory departments and participate in integrity evaluations to enhance their corporate images.

Secondly, the credibility of third-party testing agencies is crucial to the healthy development of the pharmaceutical market. Strengthening internal management and ensuring the fairness and transparency of testing processes are the keys to enhancing their international competitiveness. Refusing rent-seeking behaviors not only consolidates their own market positions but also significantly enhances the willingness of enterprises to operate with integrity. Testing agencies should regard fair testing as their mission. By actively accepting supervision, improving testing levels and service qualities, they can win market trust, avoid short-term interests harming long - term reputations, and promote the healthy growth of the global pharmaceutical industry.

In addition, the strictness and fairness of government regulators are the last line of defense for maintaining market order and public health. By optimizing the reward and punishment mechanism, they can not only encourage integrity-based operation but also effectively curb rent-seeking and dishonesty. However, although theoretical analysis shows that increasing the punishment intensity has a significant effect on suppressing rent-seeking behaviors, under the existing regulatory framework, the implementation of the reward and punishment mechanism faces multiple challenges. The limited regulatory resources restrict regulatory agencies from comprehensively monitoring illegal behaviors, especially in highly specialized and information - asymmetric fields such as the pharmaceutical industry. Excessive punishment may inhibit the innovation motivation of enterprises, while too-light punishment cannot form an effective deterrence. Therefore, when designing the reward and punishment mechanism, the government needs to balance the intensity of punishment within the legal framework, and at the same time consider industry characteristics, enterprise scales, and the nature of illegal behaviors to ensure the fairness and rationality of the mechanism. Specifically, the government should start from two aspects. Firstly, clarify and increase the economic punishment intensity for rent - seeking behaviors of pharmaceutical enterprises to make it exceed a certain threshold. By significantly increasing the amount of fines to exceed the potential benefits brought by rent-seeking behaviors, the illegal cost of enterprises can be effectively increased. For serious dishonest behaviors, the economic punishment should be sufficient to weaken the competitiveness of enterprises in the market and may even lead to enterprise bankruptcy liquidation to form a strong deterrent effect. Secondly, the government should consider implementing criminal punishments. In key areas involving public health and safety, the pursuit of criminal liability is crucial. For behaviors of serious rent-seeking or causing major damage consequences, the government can pursue the criminal liability of relevant responsible persons in accordance with the law, implement punitive detention, deprive of business qualifications and other measures, thereby enhancing the deterrence of the law. In addition, in order to effectively curb rent-seeking, the blacklist system should be strengthened, a public supervision and reporting mechanism should be established, and multi-department joint law enforcement and other measures should be considered. Although these policy tools will increase regulatory costs in the short term, in the long run, by improving regulatory effects and reducing illegal behaviors, the overall regulatory cost can be reduced, and at the same time, market confidence and stability can be enhanced, thereby reducing the costs of supervision and law enforcement.

Finally, drug wholesale enterprises, as the bridge connecting production and consumption, are crucial in maintaining market order through the reporting mechanism. Enterprises should establish and improve internal reporting and feedback mechanisms to ensure that problem products are reported quickly. The government should provide convenient reporting channels for wholesale enterprises, protect the rights and interests of whistleblowers, and give appropriate rewards to stimulate their enthusiasm for participating in supervision.

In conclusion, the government, enterprises, and third-party testing agencies need to flexibly adjust strategies according to their respective national conditions to jointly promote the construction of the integrity supervision system in the pharmaceutical industry and promote the healthy development of the global pharmaceutical market and the protection of public health. However, it is worth noting that the model construction and analysis in this study are mainly based on the game theory framework, assuming that all participants make decisions based on bounded rational behavior. This assumption simplifies the complexity of the real world to a certain extent. Therefore, although the model can reveal the effect of the reward and punishment mechanism under ideal conditions, when applied to actual scenarios, the uncertainty brought by irrational behaviors and the possibility of deviation from predicted results need to be considered. To make up for this limitation, future research can try to introduce the perspective of behavioral economics, incorporate irrational behaviors into model consideration, in order to more comprehensively reflect the decision-making process in the real world and thus improve the prediction accuracy and application value of the model."

**Comment #3:**

The model assumes rational behavior, overlooking factors like political pressure or hidden conflicts of interest that could affect stakeholder decisions.

**Response:** We sincerely thank you for your meticulous review of this article and the valuable suggestions you have put forward. This article uses evolutionary game theory to study the integrity supervision strategies of pharmaceutical enterprises. The assumed participants are of bounded rationality rather than full rationality. It is just that this article does not consider political pressure or hidden conflict of interests as a separate parameter. We understand and agree with your suggestions. Given the existence of irrational factors, we are aware of the complexity and challenges in its implementation within the existing supervision framework. We re-examine and optimize the policy recommendations to ensure that, on the basis of considering the complexity of reality, the proposed countermeasures are more practical and have stronger operability (on page 29). We added "This paper constructs an evolutionary game model for the integrity supervision of pharmaceutical enterprises, analyzes the influence of various key factors on strategy evolution, and proposes targeted decision-making suggestions for all parties. When deeply exploring the theoretical effects of the reward and punishment mechanism, we also realize the complexity and challenges of its implementation under the existing regulatory framework.

Firstly, drug production enterprises, as the cornerstone of market integrity, their behaviors are directly related to public health and industry reputation. They should actively choose integrity-based operation and establish a complete internal monitoring and compliance system to ensure product quality and safety. Especially when facing third-party testing agencies, they need to adhere to transparent cooperation and resolutely resist rent-seeking behaviors. Under the guidance of the government's reward and punishment mechanism, enterprises should strengthen communication with regulatory departments and participate in integrity evaluations to enhance their corporate images.

Secondly, the credibility of third-party testing agencies is crucial to the healthy development of the pharmaceutical market. Strengthening internal management and ensuring the fairness and transparency of testing processes are the keys to enhancing their international competitiveness. Refusing rent-seeking behaviors not only consolidates their own market positions but also significantly enhances the willingness of enterprises to operate with integrity. Testing agencies should regard fair testing as their mission. By actively accepting supervision, improving testing levels and service qualities, they can win market trust, avoid short-term interests harming long - term reputations, and promote the healthy growth of the global pharmaceutical industry.

In addition, the strictness and fairness of government regulators are the last line of defense for maintaining market order and public health. By optimizing the reward and punishment mechanism, they can not only encourage integrity-based operation but also effectively curb rent-seeking and dishonesty. However, although theoretical analysis shows that increasing the punishment intensity has a significant effect on suppressing rent-seeking behaviors, under the existing regulatory framework, the implementation of the reward and punishment mechanism faces multiple challenges. The limited regulatory resources restrict regulatory agencies from comprehensively monitoring illegal behaviors, especially in highly specialized and information - asymmetric fields such as the pharmaceutical industry. Excessive punishment may inhibit the innovation motivation of enterprises, while too-light punishment cannot form an effective deterrence. Therefore, when designing the reward and punishment mechanism, the government needs to balance the intensity of punishment within the legal framework, and at the same time consider industry characteristics, enterprise scales, and the nature of illegal behaviors to ensure the fairness and rationality of the mechanism. Specifically, the government should start from two aspects. Firstly, clarify and increase the economic punishment intensity for rent - seeking behaviors of pharmaceutical enterprises to make it exceed a certain threshold. By significantly increasing the amount of fines to exceed the potential benefits brought by rent-seeking behaviors, the illegal cost of enterprises can be effectively increased. For serious dishonest behaviors, the economic punishment should be sufficient to weaken the competitiveness of enterprises in the market and may even lead to enterprise bankruptcy liquidation to form a strong deterrent effect. Secondly, the government should consider implementing criminal punishments. In key areas involving public health and safety, the pursuit of criminal liability is crucial. For behaviors of serious rent-seeking or causing major damage consequences, the government can pursue the criminal liability of relevant responsible persons in accordance with the law, implement punitive detention, deprive of business qualifications and other measures, thereby enhancing the deterrence of the law. In addition, in order to effectively curb rent-seeking, the blacklist system should be strengthened, a public supervision and reporting mechanism should be established, and multi-department joint law enforcement and other measures should be considered. Although these policy tools will increase regulatory costs in the short term, in the long run, by improving regulatory effects and reducing illegal behaviors, the overall regulatory cost can be reduced, and at the same time, market confidence and stability can be enhanced, thereby reducing the costs of supervision and law enforcement.

Finally, drug wholesale enterprises, as the bridge connecting production and consumption, are crucial in maintaining market order through the reporting mechanism. Enterprises should establish and improve internal reporting and feedback mechanisms to ensure that problem products are reported quickly. The government should provide convenient reporting channels for wholesale enterprises, protect the rights and interests of whistleblowers, and give appropriate rewards to stimulate their enthusiasm for participating in supervision.

In conclusion, the government, enterprises, and third-party testing agencies need to flexibly adjust strategies according to their respective national conditions to jointly promote the construction of the integrity supervision system in the pharmaceutical industry and promote the healthy development of the global pharmaceutical market and the protection of public health. However, it is worth noting that the model construction and analysis in this study are mainly based on the game theory framework, assuming that all participants make decisions based on bounded rational behavior. This assumption simplifies the complexity of the real world to a certain extent. Therefore, although the model can reveal the effect of the reward and punishment mechanism under ideal conditions, when applied to actual scenarios, the uncertainty brought by irrational behaviors and the possibility of deviation from predicted results need to be considered. To make up for this limitation, future research can try to introduce the perspective of behavioral economics, incorporate irrational behaviors into model consideration, in order to more comprehensively reflect the decision-making process in the real world and thus improve the prediction accuracy and application value of the model."

**Comment #4:**

Key external factors such as globalization, technological advancements, and international regulatory variations are missing, limiting the study's scope.

**Response:** We are very grateful for your meticulous review of this article and the valuable comments you have provided. We fully understand and attach great importance to your considerations regarding key external factors such as globalization, technological progress, and international regulatory differences. These factors undoubtedly enrich the perspective of the research on the integrity supervision of pharmaceutical enterprises and provide a more comprehensive background and depth for our research.

Our research mainly focuses on the evolutionary game model involving four parties, namely drug production enterprises, third-party testing agencies, government regulators, and drug wholesale enterprises, under the reward and punishment mechanism. The aim is to deeply understand the strategy selection and evolution process of each participating party under this mechanism. By constructing and analyzing this model, we explore how to optimize the reward - punishment mechanism to improve the efficiency of integrity supervision of pharmaceutical enterprises and the influence of various key elements on strategy evolution.

We do admit that external factors such as globalization, technological progress, and international regulatory differences also have important impacts on the integrity supervision of pharmaceutical enterprises. These factors provide a rich background and depth for our research in the real world. However, in order to maintain the focus of the research, we choose to concentrate on exploring the core role of the reward - punishment mechanism in this study.

Regarding the key external factors such as globalization, technological progress, and international regulatory differences you mentioned, we fully agree that they have far-reaching impacts on the integrity supervision of pharmaceutical enterprises. In the context of globalization, the complexity of the supply chain and the competitive pressure in the international market, the innovation of regulatory means brought about by technological progress, and the differences in regulatory policies among different countries and regions are all dimensions that cannot be ignored in the integrity supervision of pharmaceutical enterprises. These factors not only affect the behavioral decisions of enterprises but also pose new challenges to the design and implementation of regulatory mechanisms.

We are very grateful for your valuable suggestions. In future research, we plan to take these key external factors into account in order to construct a more comprehensive and dynamic analytical framework. Specifically, we plan to explore how to optimize regulatory strategies in the context of the global supply chain to ensure drug quality and safety while promoting international cooperation and information sharing. How new technologies change regulatory methods and improve regulatory efficiency, and how to use big data, artificial intelligence and other means to achieve more accurate integrity supervision. How regulatory policies in different countries and regions affect enterprise behavior, and how to coordinate regulatory policies at the international level to promote global integrity standards in the pharmaceutical industry.

Thank you again for your valuable comments. We will seriously consider them and incorporate them into future research designs, hoping to make more comprehensive and in-depth contributions to the theory and practice of integrity supervision of pharmaceutical enterprises.

**Comment #5:**

Theoretical outcomes lack real-world validation through case studies or empirical data, weakening the model's practical credibility.

**Response:** We sincerely thank you for your detailed review and valuable suggestions on this article. We fully understand and highly respect your concern that the theoretical results lack verification in the real world through case studies or empirical data, which indeed poses a reasonable challenge to the practical credibility of the model. The gap between theoretical analysis and real-world verification pointed out by you is a major challenge in our research.

When setting the model parameters, we referred to officially released data as much as possible, such as "2022 Annual Data of Drug Supervision and Administration Statistics", "2023 Department Budget of the National Medical Products Administration", "FDA Annual Performance and Financial Report", "Overview of Health Economic Data in the UK" and "World Health Statistics", to ensure the rationality of key parameters such as the cost of drug production enterprises producing low - quality drugs and the cost of strict government supervision. However, we also recognize that the setting of some parameters depends on literature reviews and comprehensive analysis of the current situation of the industry, which does limit the empirical support of the model to a certain extent.

This research aims to construct a theoretical framework to explore the role of the government's reward and punishment mechanism in the integrity supervision of pharmaceutical enterprises. Given the concealment of rent-seeking behavior and the sensitivity of data, there are indeed objective difficulties in obtaining detailed empirical data. Our strategy is to set parameters based on existing literature and the current situation of the industry to provide a preliminary analytical perspective.

Nevertheless, we fully understand and agree with your view that the supplementation of empirical data is crucial for enhancing the practicality and credibility of the model. Given the limitations of the current research, we plan to strengthen the empirical basis of the model in subsequent research by introducing more case studies, empirical data analysis and industry surveys. At the same time, we also consider cooperating with industry experts to obtain more direct industry data and insights, and further verify the fit between model predictions and industry practices.

Here, we sincerely ask the reviewers to understand that, given the nature and conditional limitations of the current research, we are temporarily unable to conduct a comprehensive empirical verification of the model. We promise that in future research, we will focus on strengthening the support of empirical data to enhance the practicality and influence of the research.

We are very grateful for the in-depth feedback you provided on this article, which will greatly promote our future research. We look forward to being able to better meet your expectations for empirical verification in subsequent work and provide more solid support for the theoretical research and policy-making of integrity supervision of pharmaceutical enterprises. Thank you again for your valuable suggestions. Your guidance will have a far-reaching impact on our research.

**Comment #6:**

Policy recommendations are too broad and vague, particularly regarding reducing supervision costs and increasing penalties for rent-seeking behavior. More detailed, actionable policy tools and cost-benefit analyses are required.

**Response:** Thank you very much for your suggestions. As you suggested, we added more specific suggestions (on page 29). We added "This paper constructs an evolutionary game model for the integrity supervision of pharmaceutical enterprises, analyzes the influence of various key factors on strategy evolution, and proposes targeted decision-making suggestions for all parties. When deeply exploring the theoretical effects of the reward and punishment mechanism, we also realize the complexity and challenges of its implementation under the existing regulatory framework.

Firstly, drug production enterprises, as the cornerstone of market integrity, their behaviors are directly related to public health and industry reputation. They should actively choose integrity-based operation and establish a complete internal monitoring and compliance system to ensure product quality and safety. Especially when facing third-party testing agencies, they need to adhere to transparent cooperation and resolutely resist rent-seeking behaviors. Under the guidance of the government's reward and punishment mechanism, enterprises should strengthen communication with regulatory departments and participate in integrity evaluations to enhance their corporate images.

Secondly, the credibility of third-party testing agencies is crucial to the healthy development of the pharmaceutical market. Strengthening internal management and ensuring the fairness and transparency of testing processes are the keys to enhancing their international competitiveness. Refusing rent-seeking behaviors not only consolidates their own market positions but also significantly enhances the willingness of enterprises to operate with integrity. Testing agencies should regard fair testing as their mission. By actively accepting supervision, improving testing levels and service qualities, they can win market trust, avoid short-term interests harming long - term reputations, and promote the healthy growth of the global pharmaceutical industry.

In addition, the strictness and fairness of government regulators are the last line of defense for maintaining market order and public health. By optimizing the reward and punishment mechanism, they can not only encourage integrity-based operation but also effectively curb rent-seeking and dishonesty. However, although theoretical analysis shows that increasing the punishment intensity has a significant effect on suppressing rent-seeking behaviors, under the existing regulatory framework, the implementation of the reward and punishment mechanism faces multiple challenges. The limited regulatory resources restrict regulatory agencies from comprehensively monitoring illegal behaviors, especially in highly specialized and information - asymmetric fields such as the pharmaceutical industry. Excessive punishment may inhibit the innovation motivation of enterprises, while too-light punishment cannot form an effective deterrence. Therefore, when designing the reward and punishment mechanism, the government needs to balance the intensity of punishment within the legal framework, and at the same time consider industry characteristics, enterprise scales, and the nature of illegal behaviors to ensure the fairness and rationality of the mechanism. Specifically, the government should start from two aspects. Firstly, clarify and increase the economic punishment intensity for rent-seeking behaviors of pharmaceutical enterprises to make it exceed a certain threshold. By significantly increasing the amount of fines to exceed the potential benefits brought by rent-seeking behaviors, the illegal cost of enterprises can be effectively increased. For serious dishonest behaviors, the economic punishment should be sufficient to weaken the competitiveness of enterprises in the market and may even lead to enterprise bankruptcy liquidation to form a strong deterrent effect. Secondly, the government should consider implementing criminal punishments. In key areas involving public health and safety, the pursuit of criminal liability is crucial. For behaviors of serious rent-seeking or causing major damage consequences, the government can pursue the criminal liability of relevant responsible persons in accordance with the law, implement punitive detention, deprive of business qualifications and other measures, thereby enhancing the deterrence of the law. In addition, in order to effectively curb rent-seeking, the blacklist system should be strengthened, a public supervision and reporting mechanism should be established, and multi-department joint law enforcement and other measures should be considered. Although these policy tools will increase regulatory costs in the short term, in the long run, by improving regulatory effects and reducing illegal behaviors, the overall regulatory cost can be reduced, and at the same time, market confidence and stability can be enhanced, thereby reducing the costs of supervision and law enforcement.

Finally, drug wholesale enterprises, as the bridge connecting production and consumption, are crucial in maintaining market order through the reporting mechanism. Enterprises should establish and improve internal reporting and feedback mechanisms to ensure that problem products are reported quickly. The government should provide convenient reporting channels for wholesale enterprises, protect the rights and interests of whistleblowers, and give appropriate rewards to stimulate their enthusiasm for participating in supervision.

In conclusion, the government, enterprises, and third-party testing agencies need to flexibly adjust strategies according to their respective national conditions to jointly promote the construction of the integrity supervision system in the pharmaceutical industry and promote the healthy development of the global pharmaceutical market and the protection of public health. However, it is worth noting that the model construction and analysis in this study are mainly based on the game theory framework, assuming that all participants make decisions based on bounded rational behavior. This assumption simplifies the complexity of the real world to a certain extent. Therefore, although the model can reveal the effect of the reward and punishment mechanism under ideal conditions, when applied to actual scenarios, the uncertainty brought by irrational behaviors and the possibility of deviation from predicted results need to be considered. To make up for this limitation, future research can try to introduce the perspective of behavioral economics, incorporate irrational behaviors into model consideration, in order to more comprehensively reflect the decision-making process in the real world and thus improve the prediction accuracy and application value of the model."

**Comment #7:**

The model would benefit from real-world scenario discussions, providing clearer guidance for policymakers.

**Response:** Thank you very much for your suggestions. We agree with your point of view that real-world scenario discussions should be provided for the model. Therefore, we added the 3.1 Problem Description part in the "3 Model Hypotheses and Construction" section (the second paragraph on page 6). The specific content is as follows: "In the context of globalization, the pharmaceutical industry is facing increasingly complex regulatory challenges. In particular, rent-seeking behavior erodes the integrity supervision system, threatening public health and market fairness. How to promote the integrity of pharmaceutical enterprises and build a fair and transparent market environment through effective reward and punishment mechanisms has become a key issue that urgently needs to be addressed. In response to this real-world scenario, this paper constructs an evolutionary game model involving drug production enterprises, third-party testing agencies, government regulators and drug wholesale enterprises. Through model analysis, we aim to gain a deeper understanding of the strategy choice and evolution process of each participating party under the reward and punishment mechanism, and explore how to optimize the reward and punishment mechanism to improve the efficiency of integrity supervision of pharmaceutical enterprises. This paper will provide policy makers with decision-making bases based on real market behaviors, guiding the government, enterprises and third-party agencies on how to formulate and implement more effective reward and punishment policies in specific situations, in order to promote integrity operation, maintain market order, safeguard public health, and build a harmonious pharmaceutical market environment. Therefore, the construction and analysis of this model will directly benefit from in-depth exploration of specific real-world scenarios, providing policy makers with clearer and more practical guiding directions."

**Comment #8:**

Extending the model to include non-rational behavior, international influences, or corruption would offer a more realistic representation of pharmaceutical regulation.

**Response:** We are very grateful for the valuable comments put forward by the reviewers. The complex factors such as irrational behavior, international influence and corruption mentioned by you can indeed add more practical significance to our model. In the current research, we mainly focus on the game analysis under bounded rational behavior. However, in the future, we plan to incorporate these factors into the model to more comprehensively reflect the complexity of pharmaceutical supervision. We deeply feel the importance of these directions for improving the research quality and practical application value, and will fully consider them in the subsequent research. Thank you again for your guidance and suggestions, and we look forward to pushing the research to a new height.

**Comment #9:**

Case studies or empirical data from various regulatory environments would help validate the model’s predictions and identify necessary refinements.

**Response:** Thank you very much for your valuable comments and suggestions. We fully understand and agree that the introduction of case studies or empirical data in different regulatory environments will more effectively verify the predictive ability of the model and identify possible optimization points.

In the current research, the model parameter setting and verification are mainly based on existing official data, literature review, and industry situation analysis. We plan to further collect and analyze detailed cases from different global pharmaceutical regulatory environments in subsequent research to enrich and refine our research. This will not only help improve the applicability and prediction accuracy of the model but also provide more solid empirical support for our theoretical framework, ensuring the extensiveness and practicality of the research results.

We are deeply grateful for the professional guidance of the reviewers and will actively adopt and implement this suggestion, hoping to present more comprehensive and in - depth analysis results in subsequent research.

**Comment #10:**

Recommendations on penalties for rent-seeking should explore specific forms (financial, criminal) and enforcement methods.

**Response:** Thank you for your suggestions. We have carried out a more specific discussion on the suggestions for rent-seeking penalties (on page 30). We have revised it as "In addition, the strictness and fairness of government regulators are the last line of defense for maintaining market order and public health. By optimizing the reward and punishment mechanism, they can not only encourage integrity-based operation but also effectively curb rent-seeking and dishonesty. However, although theoretical analysis shows that increasing the punishment intensity has a significant effect on suppressing rent-seeking behaviors, under the existing regulatory framework, the implementation of the reward and punishment mechanism faces multiple challenges. The limited regulatory resources restrict regulatory agencies from comprehensively monitoring illegal behaviors, especially in highly specialized and information-asymmetric fields such as the pharmaceutical industry. Excessive punishment may inhibit the innovation motivation of enterprises, while too-light punishment cannot form an effective deterrence. Therefore, when designing the reward and punishment mechanism, the government needs to balance the intensity of punishment within the legal framework, and at the same time consider industry characteristics, enterprise scales, and the nature of illegal behaviors to ensure the fairness and rationality of the mechanism. Specifically, the government should start from two aspects. Firstly, clarify and increase the economic punishment intensity for rent-seeking behaviors of pharmaceutical enterprises to make it exceed a certain threshold. By significantly increasing the amount of fines to exceed the potential benefits brought by rent-seeking behaviors, the illegal cost of enterprises can be effectively increased. For serious dishonest behaviors, the economic punishment should be sufficient to weaken the competitiveness of enterprises in the market and may even lead to enterprise bankruptcy liquidation to form a strong deterrent effect. Secondly, the government should consider implementing criminal punishments. In key areas involving public health and safety, the pursuit of criminal liability is crucial. For behaviors of serious rent-seeking or causing major damage consequences, the government can pursue the criminal liability of relevant responsible persons in accordance with the law, implement punitive detention, deprive of business qualifications and other measures, thereby enhancing the deterrence of the law. In addition, in order to effectively curb rent-seeking, the blacklist system should be strengthened, a public supervision and reporting mechanism should be established, and multi-department joint law enforcement and other measures should be considered. Although these policy tools will increase regulatory costs in the short term, in the long run, by improving regulatory effects and reducing illegal behaviors, the overall regulatory cost can be reduced, and at the same time, market confidence and stability can be enhanced, thereby reducing the costs of supervision and law enforcement."

**Comment #11:**

Ethical implications of rent-seeking and the role of corporate governance should be discussed to offer a more comprehensive approach to integrity.

**Response:** Thank you for your suggestions. According to your suggestions, we added the definition of rent-seeking behavior in the "1 Introduction" part, and analyzed the practical impacts including ethical significance and the role of enterprise supervision (the first paragraph on page 2). The specific content is "The integrity of pharmaceutical enterprises is an embodiment of national governance capabilities and an important guarantee for the healthy development of the economy and society. The lack of integrity in the pharmaceutical field seriously affects the normal operation of the medical industry, especially when rent-seeking behaviors are involved. Rent-seeking behavior refers to the fact that enterprises or individuals obtain economic benefits through non-market means, such as bribery or interest transfer, rather than achieving profit growth through improving efficiency or innovation. In the pharmaceutical industry, drug production enterprises influence the test results of third-party testing agencies through rent-seeking behavior, which not only distorts normal medical behaviors and reduces the quality of medical services, but also may allow unqualified drugs to enter the market, seriously threatening the life safety of patients. From an ethical perspective, rent-seeking behavior places economic interests above patients' health and is a betrayal of professional spirit and social responsibility. At the same time, rent-seeking behavior leads to unfair allocation of medical resources, exacerbates excessive medical treatment and drug price increases, increases the economic burden on patients, and ultimately hinders the healthy and sustainable development of the pharmaceutical industry. Against this background, it is urgent to strengthen corporate governance to ensure that corporate behaviors comply with ethical norms and legal regulations, thereby reshaping industry trust and promoting a virtuous cycle in the pharmaceutical industry."

We have tried our best to improve the manuscript and made some substantial changes and necessary deletions according to the editors’ and reviewers’ comments. We earnestly appreciate the editors’ and reviewers’ professional work and hope that the corrections will make our manuscript suitable for publication in *PLOS ONE*. We are looking forward to receiving comments from reviewers in the future. If you have any questions, please do not hesitate to contact me at the address below.

Once again, thank you very much for your valuable comments and suggestions.

Best wishes.

Sincerely,

Lilong Zhu, Ph.D. and Professor. Postdoctoral research in Shandong University. Research scholar in College of business, University of Illinois at Urbana-Champaign, USA. Ph.D. degree was granted in management science and engineering from Tongji University and Postdoctoral research in Shandong University. His research interests are supply chain management and quality management.

E-mail: [*zhulilong2008@126.com*](mailto:zhulilong2008@126.com) Tel: +86-13853193366

College of Business, Shandong Normal University, School of Management, Shandong University, Ji’nan 250014, Shandong, China.
