# Supplementary material for: Pharmaceutical enterprises integrity supervision strategy when considering rent-seeking behavior and government reward and punishment mechanism
Source: PLoS One. 2025 May 19;20(5):e0320964. doi: 10.1371/journal.pone.0320964 (PMC12088600; doi:10.1371/journal.pone.0320964)
Supplement: Attachment — Submitted filename: Response_to_Reviewers_auresp_2.docx [file pone.0320964.s004.docx]

**Responses to Editor and** **Reviewers**

**Dear Editor Professor** **Helen Howard and Reviewers,**

Thank you very much for giving us an opportunity to submit a revised version of the manuscript entitled “*Pharmaceutical Enterprises Integrity Supervision Strategy When Considering Rent-seeking Behavior and Government Reward and Punishment Mechanism*.” The manuscript is a revised submission (Manuscript ID: ***PONE-D-24-28268***) with new page numbers in the text, some grammar and spelling errors have also been corrected. The revised paper contains 36 pages, 10 figures and 4 tables.

We also thank the reviewers for their critical reading and the valuable feedback from the peer reviewers. These comments provide directions for our further research. According to the reviewers' opinions, the original manuscript was modified accordingly, and the main modifications were marked with different colors. We will mark the modification of the first opinion in blue, and the modification of the second opinion in red, and reply to each opinion listed below one by one, and clearly point out the location of the revision.

**Responds to the editor ’s comments:**

**Comment #1:**

Please work to improve the quality of the writing throughout your manuscript. We recommend asking a colleague who is proficient in written English to assist you; alternatively, you could enlist the help of a professional copyediting service.

**Response**: Thank you very much for your suggestion. We are well aware of the importance of accurate expression to the quality of academic papers. We have carried out a detailed grammar check and revision of the full text to improve the readability and professionalism of the paper, and have made some modifications to improve the writing quality of the manuscript and strive to make the original manuscript more excellent.

**Comment #2:**

We note that you have published a paper with a similar methodology and focus in the journal Complexity (https://onlinelibrary.wiley.com/doi/10.1155/2021/5865299). Given the similarity in some of the hypotheses between this paper and your current submission, it needs to be made clear how these two studies differ in terms of the research questions being asked and the current study framed within the context of the literature. Please clarify this in either the covering letter accompanying the revised version or within the text itself.

**Response**: Thank you for your careful review and valuable comments. In the materials currently submitted, we have carefully considered your concerns. The research of Zhang and zhu[11] mainly focused on the collaborative supervision between local governments, pharmaceutical enterprises and third-party testing agencies. They focused on how to improve the integrity management level of pharmaceutical enterprises through the participation of third-party testing agencies under the government reward and punishment mechanism. Their research focuses on how to promote the honest operation of pharmaceutical enterprises and reduce the flow of unqualified drugs into the market through the synergy of local governments and third-party testing agencies. On the basis of Zhang and Zhu, the current research further introduces the role of pharmaceutical wholesale enterprises, and constructs a four parties evolutionary game model including pharmaceutical enterprises, third-party testing agencies, government regulators and pharmaceutical wholesale enterprises. Our research questions not only include how to promote the honest operation of pharmaceutical enterprises through the government reward and punishment mechanism, but also pay special attention to the impact of drug wholesale enterprises' reporting behavior on the strategic choice of pharmaceutical enterprises and third-party testing agencies. In addition, we also discussed how to improve the efficiency of government regulation and promote the integrity level of the entire drug supply chain by optimizing the reward and punishment mechanism.

We have added relevant explanations on page 5 of the original text. The specific content is "The research by Zhang and Zhu [11] has provided valuable insights into the co-regulation of pharmaceutical enterprises. However, their study has certain limitations. Firstly, their research only considered the relationships among local governments, drug enterprises, and third-party testing agencies, while ignoring the important role of drug wholesale enterprises in the supervision of drug quality. Secondly, their model assumptions are relatively simple and do not fully take into account the whistleblowing behavior of drug wholesale enterprises and its impact on the strategic choices of all parties. Compared with their study, this research introduces drug wholesale enterprises as a new participant and constructs a more complex four-party evolutionary game model, which can more comprehensively reflect the actual game relationship in the pharmaceutical supply chain. " We hope these clarifications can address your concerns. Thank you again for your time and attention.

We have tried our best to improve the manuscript and made some substantial changes and necessary deletions according to the editors’ and reviewers’ comments. We earnestly appreciate the editors’ and reviewers’ professional work and hope that the corrections will make our manuscript suitable for publication in *PLOS ONE*. We are looking forward to receiving comments from reviewers in the future. If you have any questions, please do not hesitate to contact me at the address below.

Once again, thank you very much for your valuable comments and suggestions.

Best wishes.

Sincerely,

Lilong Zhu, Ph.D. and Professor. Postdoctoral research in Shandong University. Research scholar in College of business, University of Illinois at Urbana-Champaign, USA. Ph.D. degree was granted in management science and engineering from Tongji University and Postdoctoral research in Shandong University. His research interests are supply chain management and quality management.

E-mail: [*zhulilong2008@126.com*](mailto:zhulilong2008@126.com) Tel: +86-13853193366

College of Business, Shandong Normal University, School of Management, Shandong University, Ji’nan 250014, Shandong, China.
